# CircORC2 promoted proliferation and inhibited the sensitivity of osteosarcoma cell lines to cisplatin by regulating the miR‐485‐3p/TRIM2 axis

**DOI:** 10.1002/ccs3.12029

**Published:** 2024-04-25

**Authors:** Tianhua Chen, Zuyang Zhang, Chao Tian, Yuchao Feng, Xiaojie He, Liangdong Jiang

**Affiliations:** ^1^ The Affiliated Changsha Central Hospital Department of Orthopaedics Hengyang Medical School University of South China Changsha Hunan Province China; ^2^ Laboratory of Pediatric Nephrology Institute of Pediatrics Central South University Changsha Hunan Province China

**Keywords:** circORC2, cisplatin resistance, MiR‐485‐3p, osteosarcoma, TRIM2

## Abstract

Resistance to chemotherapy leads to poor prognosis for osteosarcoma (OS) patients. However, due to the high metastasis of tumor and the decrease in sensitivity of tumor cells to cisplatin (DDP), the 5‐year survival rate of OS patients is still unsatisfactory. This study explored a mechanism for improving the sensitivity of OS cells to DDP. A DDP‐resistant OS cell model was established, and we have found that circORC2 and TRIM2 were upregulated in DDP‐resistant OS cells, but miR‐485‐3p was downregulated. The cell viability and proliferation of the OS cells decreased gradually with the increase of DDP dose, but a gradual increase in apoptosis was noted. CircORC2 promoted OS cell proliferation and DDP resistance and upregulated TRIM2 expression by targeting miR‐485‐3p. Functionally, circORC2 downregulated miR‐485‐3p to promote OS cell proliferation and inhibit DDP sensitivity. Additionally, it promoted cell proliferation and inhibited the sensitivity of DDP by regulating the miR‐485‐3p/TRIM2 axis. In conclusion, circORC2 promoted cell proliferation and inhibited the DDP sensitivity in OS cells via the miR‐485‐3p/TRIM2 axis. These findings indicated the role of circORC2 in regulating the sensitivity of OS cells to DDP.

## Graphical abstract

CircORC2 sponges miR‐485‐3p and upregulates TRIM2 to promote the osteosarcoma cell lines proliferation and cisplatin chemotherapy resistance.

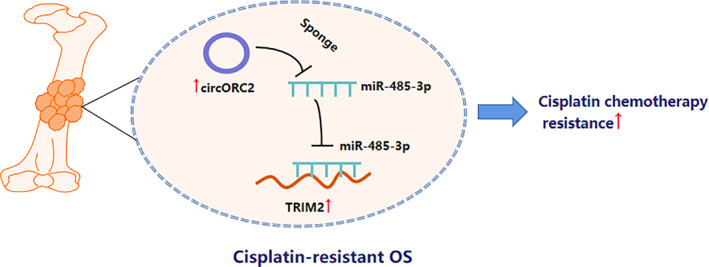

## 1 INTRODUCTION

Osteosarcoma (OS) is the most common primary solid malignancy characterized by malignant mesenchymal cells that produce bone‐like substances or immature bone.^1^ This disease commonly affects adolescents between the ages of 15 and 19.^2^ OS has a high degree of metastasis with high rates of disability, mortality, and recurrence, leading to a poor prognosis.^3^ The main treatment strategies for OS over the past few decades include surgery, chemotherapy, radiotherapy, and immunotherapy. However, due to the high metastasis and recurrence rates, coupled with the emergence of drug resistance, the prognosis of OS patients is poor. Therefore, chemoresistance and metastatic spread are the major clinical problems in OS patients.^4^ Cisplatin (cis‐diamminedichloroplatinum, DDP) is the most common anticancer drug used for OS, but the decrease in the sensitivity of OS cells to DDP due to drug resistance poses a challenge in OS therapy.^5^ There is an urgent need to understand the mechanism for improving the sensitivity of tumor cells to DDP and the effectiveness of targeted therapies to improve overall survival in patients with OS.

Circular RNAs (circRNAs) are competing endogenous RNAs (ceRNAs) characterized by a closed continuous loop structure without a 5′ cap and a poly(A) tail at the 3′ ends.^6^ The main function of circRNAs is to act as a molecular sponge. They can target and inhibit the transcription of miRNAs, thus enhancing the expression of the target gene. circRNA plays a regulatory role in different diseases via different mechanisms.^7^ Most circRNAs are highly expressed in OS, and carcinogenic circRNAs can accelerate OS progression, the carcinogenic ability of circRNA is mainly reflected in promoting cell proliferation and metastasis and affecting apoptosis.^8^ Circ‐0001658 promotes proliferation and metastasis and blocks apoptosis in OS cells by sponging miR‐382‐5p and regulating the expression of YB‐1.^9^ According to Wu et al., circTADA2A overexpression promotes metastasis in OS by sponging miR‐203a‐3p.^10^ CircORC2 is a relatively new circRNA, and recent studies have shown that it is generally upregulated in OS cell lines, promoting OS cell growth and invasion by regulating the miR‐19a/PTEN axis.^11^ However, the underlying molecular mechanism involved in OS progression remains unclear; the regulation of circORC2 and its downstream factors need to be explored.

MicroRNAs (miRNAs) are a class of non‐coding small RNAs with a length of about 20 bp.^12^ There is growing evidence that an abnormal expression of miRNAs is strongly associated with malignant progression in OS. Du et al. showed that miR‐485‐3p was upregulated in OS cells, and miR‐485‐3p overexpression controlled the malignant proliferation by inhibiting the level of CTBP1 in OS.^13^ Low levels of miR‐485‐3p were reported to inhibit cell proliferation by inhibiting the c‐MET and AKT3/mTOR signaling pathways in OS.^14^ Furthermore, circRNAs regulate miR‐485‐3p, which plays a role in regulating the progression of OS. For instance, the miR‐485‐3p/JAG1 axis is regulated by circ_0084582 to promote OS cell proliferation, metastasis, and angiogenesis.^15^ We through the starBase prediction revealed the presence of interactions between circORC2 and miR‐485‐3p. Additionally, miR‐485‐3p is known to be associated with drug resistance to chemotherapy; Zhao et al. indicated that circ_0000338 enhanced 5‐fluorouracil resistance in colorectal cancer by regulating miR‐485‐3p.^16^ However, the specific molecular mechanisms involved in regulating the sensitivity of OS cells to DDP by miR‐485‐3p and whether miR‐485‐3p is regulated by circORC2 in OS cells remain unclear. miRNAs induce target mRNA degradation or translation inhibition by pairing with complementary bases in the 3′‐untranslated region (3′‐UTR) of the target mRNA.^17^

The tripartite motif (TRIM) family consists of more than 70 members, including a ring domain, a B‐box motif, and a coiled region.^18^ TRIM‐containing protein 2 (TRIM2) is a member of the TRIM protein family and is a cyclic E3 ubiquitin ligase. It reportedly regulates tumor progression, cell biological activity, inflammation, immunity, and chemoresistance.^19^, ^20^ TRIM2 is an oncogene highly expressed in many tumors, such as ovarian, breast, pancreatic, and OS.^21^, ^22^, ^23^, ^24^ Studies have shown that TRIM2 is highly expressed in tamoxifen‐resistant MCF‐7R cells and is associated with tamoxifen resistance.^23^ Notably, TRIM2 regulates the proliferation and metastasis of OS cells. However, whether TRIM2 regulates the resistance of OS cells to DDP chemotherapy remains unclear.^22^ The starBase prediction indicated a targeted binding site between miR‐485‐3p and TRIM2. However, whether miR‐485‐3p regulates the sensitivity of OS cells to DDP through TRIM2 remains unknown.

This study investigated the regulatory effect of circORC2 on cell proliferation, the sensitivity of OS cells to DDP, and the underlying molecular mechanisms involved. We aimed to determine whether circORC2 regulates cell proliferation and the sensitivity of OS cells to DDP via the miR‐485‐3p/TRIM2 axis, thus providing a novel direction for OS clinical therapeutics.

## 2 MATERIALS AND METHODS

### 2.1 Cell lines and culture

Human osteoblast cells, HFOB1.19 (CRL‐3602), and OS cell lines, including Saos‐2 (HTB‐85), SW1353 (HTB‐94), U‐2Os (HTB‐96), SJSA‐1(CRL‐2098) and HOS (CRL‐1543), were provided by American Type Culture Collection. The cells were cultivated in Dulbecco's Modified Eagle's Medium (ThermoFisher Scientific) containing 10% fetal bovine serum and 1% P/S and incubated inside a humid incubator under 37°C temperature and 5% CO_2_ conditions.

### 2.2 Cell transfection and treatment

The oe‐circORC2 (circORC2‐pcDNA3.1), sh‐circORC2 (sh‐NC), and miR‐485‐3p mimics/inhibitor (mimics/inhibitor NC) were synthesized by GeneChem (Shanghai, China). Whole sequences of TRIM2 were generated by PCR and inserted into the pcDNA3.1 vector (GenePharma) for TRIM2 overexpression. The segments were transfected into U‐2Os and SJSA‐1 cells using Lipofectamine™ 3000 Transfection Reagent (Invitrogen, California, USA). For the cell treatment, U‐2Os and SJSA‐1 cells were exposed to various concentrations (0, 1, 2, 4, and 8 μM) of DDP (Yuanyebio, Shanghai, 15663‐27‐1).

### 2.3 Quantitative reverse transcriptase‐polymerase chain reaction (qRT‐PCR)

All reagents and commercial kits were purchased from Invitrogen. Total RNA was extracted from the U‐2Os and SJSA‐1 cells using the TRIzol reagent. The PrimeScript RT Reagent Kit was used to reverse transcribe 1 μg of total RNA into complementary DNA (cDNA). The miRNAs were collected using mirVana microRNA Isolation kits. The Taqman microRNA assay kit was used to detect the miR‐485‐3p level (U6 RNA served as endogenous control). The mRNA levels of circORC2 and TRIM2 were measured on a 7500 real‐time PCR system using SYBR Premix Ex Taq. GAPDH was used as an endogenous control for data analysis. The change in RNA expression was calculated using the 2^−ΔΔCT^ method. The primers used in this study are listed in Table 1.

**TABLE 1:** The primer sequences used in this study.

Primer name	Primer sequences
F‐circRNA ORC2	5′‐GATCCCTGCCACTTAGCTCC ‐3′
R‐circRNA ORC2	5′‐TCACTGTCAGAATGAGACCTTGG ‐3′
F‐miR‐485‐3p	5′‐GCCGAGGUCAUACACGGCUCU‐3′
R‐miR‐485‐3p	5′‐CTCAACTGGTGTCGTGGA‐3′
F‐TRIM2	5′‐AGGACA AAGACGGTGAGCTG ‐3′
R‐TRIM2	5′‐CTCTTCACGCCT TCTGTGGT ‐3′
F‐U6	5′‐CTCGCTTCGGCAGCACA‐3′
R‐U6	5′‐AACGCTTCACGAATTTGCGT‐3′
F‐GAPDH	5′‐CTGACTTCAACAGCGACACC‐3′
R‐GAPDH	5′‐GTGGTCCAGGGGTCTTACTC ‐3′

### 2.4 Western blot analysis

U‐2Os and SJSA‐1 cell lysates were prepared using RIPA lysis buffer (ThermoFisher Scientific). The proteins were transferred to a PVDF membrane after isolation with a 10% SDS‐PAGE gel. The PVDF membranes were blocked with TBST and 5% nonfat dry milk and incubated with specific anti‐TRIM2 antibody (ab3942; 1:1000) overnight at 4°C. GAPDH (ab8245; 1:2000) was used as an endogenous control. All antibodies were purchased from Abcam (Cambridge, UK). On the second day, after incubation with goat anti‐rabbit IgG H&L antibody (ab254262; 1:2000), the bands were detected using a GEL imaging system (Bio‐Rad, California, USA), and protein quantitative analysis was performed using the Image J software.

### 2.5 MTS assay

The MTS reagent (Saint‐Bio) was dissolved at room temperature (or 37°C), and 10 μL was added to a 96‐well culture plate containing 1 × 10^5^ cells/well. The plate was returned to the incubator and allowed to rest for 1 h at 37°C. The optical density value at 490 nm was determined using a microplate reader.

### 2.6 Colony formation assay

Single‐cell suspensions of U‐2Os and SJSA‐1 cells were seeded into 15 mm dishes, and the cell proliferation ability was determined, as described previously.^25^

### 2.7 Flow cytometry

After centrifugation, the U‐2Os and SJSA‐1 cells were resuspended with binding buffer and adjusted to 1 × 10^6^ cells/mL. Subsequently, 100 μL of the cell suspension was treated with 5 μL of Annexin V‐FITC and 5 μL of PI solution (BD Biosciences). Finally, the suspension was loaded into a flow cytometer for automatic computer analysis.

### 2.8 Bioinformatics and dual‐luciferase assay

The potential binding sites of circORC2 or miR‐485‐3p were predicted using the starBase bioinformatics software. Plasmid construction and luciferase activity were performed as described previously.^26^

### 2.9 Statistical analysis

The mean ± standard deviation (SD) represents data from three independent experiments. The Student's *t*‐test was used for two‐group comparisons, and Tukey's multiple comparison test was used for multi‐group comparisons. A *p*‐value of <0.05 was considered statistically significant.

## 3 RESULTS

### 3.1 CircORC2 and TRIM2 were upregulated, but miR‐485‐3p was downregulated in the OS cells

**FIGURE 1:**
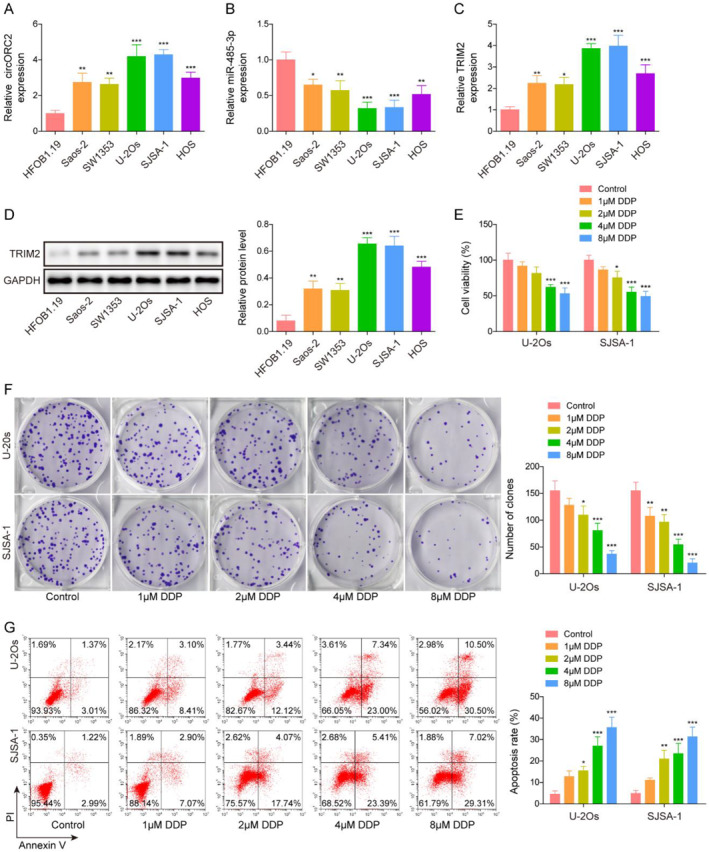
CircORC2 and TRIM2 were upregulated, but miR‐485‐3p was downregulated in OS cells. (A). Quantitative reverse transcriptase‐polymerase chain reaction (qRT‐PCR) was used to detect the circORC2 level in OS (Saos‐2, SW1353, U‐2Os, SJSA‐1, and HOS) and HFOB1.19 cells. (B). miR‐485‐3p level in OS cells assessed by qRT‐PCR. (C). QRT‐PCR was used to detect the TRIM2 mRNA levels in OS cells. (D). Western blot was employed to measure the TRIM2 protein level in OS cells. U‐2Os and SJSA‐1 cells were exposed to DDP at various concentrations (0, 1, 2, 4, and 8 μM). (E). MTS assay was used to detect the cell viability. (F). Cell proliferation was assessed using the colony formation assay. (G). Flow cytometry was used to detect cell apoptosis. Data are presented as mean ± SD of three replicate experiments (*n* = 3), *p < 0.05, **p < 0.01 and ***p < 0. 001.

CircORC2 and TRIM2 have been reported to promote the progression of OS,^11^, ^22^ but the specific mechanism involved remains unknown. Therefore, we predicted the downstream targets for circORC2 and the miRNAs that can regulate TRIM2 using the Circinteractome and starBase databases, respectively. As shown in Figure S1A, the target genes of circORC2 (miR‐217, miR‐409‐3p, miR‐485‐3p, and miR‐671‐5p) have binding sites with TRIM2 and may be the intermediate molecules connecting circORC2 with TRIM2 in OS. We further detected the expression of the above miRNAs in osteoblast (HFOB1.19) and OS cell lines; miR‐485‐3p was significantly downregulated compared to the other miRNAs (Figure S1B). circORC2 and TRIM2 were upregulated in the OS cell lines, whereas miR‐485‐3p was downregulated (Figure 1A–C). Western bot analysis demonstrated a high level of TRIM2 protein in the OS cell lines (Figure 1D). Changes in gene expression were more evident in the U‐2Os and SJSA‐1 cells; therefore, these two cell lines were used for subsequent functional experiments. The cell viability and proliferation of the U‐2Os and SJSA‐1 cells decreased gradually with the increase in the DDP dose; however, a gradual increase in apoptosis was noted (Figure 1E–G). Additionally, 4 μM of DDP significantly affected the viability of OS cells; hence, this concentration was selected for the following experiment.

### 3.2 CircORC2 promoted OS cell proliferation and DDP resistance

**FIGURE 2:**
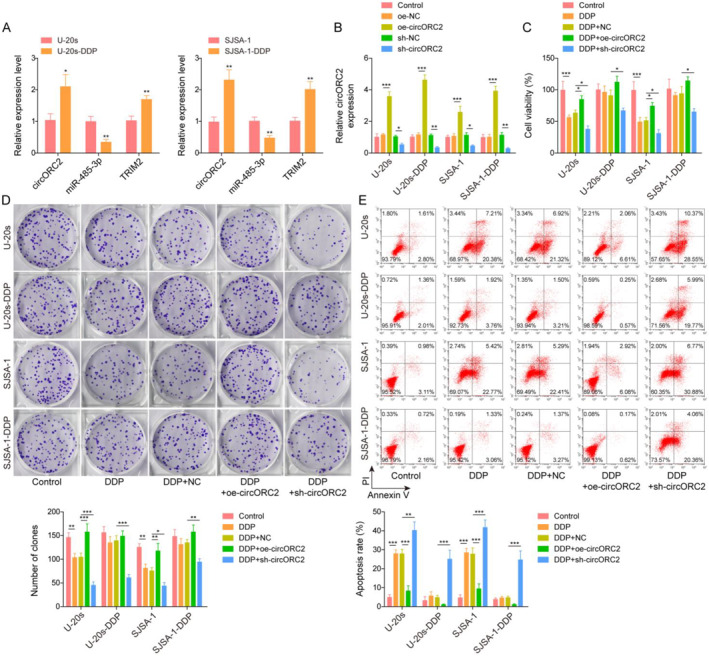
CircORC2 promoted OS cells proliferation and DDP resistance. (A). Quantitative reverse transcriptase‐polymerase chain reaction (qRT‐PCR) detected circORC2, miR‐485‐3p, and TRIM2 levels in the U‐2Os, U‐2Os‐DDP, SJSA‐1, and SJSA‐1‐DDP cells. (B). U‐2Os, U‐2Os‐DDP, SJSA‐1, and SJSA‐1‐DDP cells were transfected with oe‐circORC2 or sh‐circORC2, and qRT‐PCR was used to estimate the circORC2 expression levels in these cells. Subsequently, the cells were exposed to DDP (concentration, 4 μM). (C). The MTS assay was performed to assess the cell viability. (D). Cell proliferation was evaluated using the colony formation assay. (E). Flow cytometry was employed to assess cell apoptosis. Data are presented as mean ± SD of three replicate experiments (*n* = 3), *p < 0.05, **p < 0.01 and ***p < 0. 001.

U‐2Os and SJSA‐1 cells were exposed to DDP to construct DDP‐resistant OS cell lines (U‐2Os‐DDP and SJSA‐1‐DDP). circORC2 and TRIM2 levels were significantly upregulated but the miR‐485‐3p level was downregulated in the U‐2Os‐DDP and SJSA‐1‐DDP cells compared to those in the U‐2Os and SJSA‐1 cells (Figure 2A). Subsequently, we overexpressed and knocked down circORC2 in OS and OS DDP‐resistant cell lines (Figure 2B) and then administrated DDP. Functional experiments showed that overexpression of circORC2 promoted cell viability and proliferation, whereas circORC2 knockdown inhibited these properties in the DDP‐sensitive and resistant OS cells (Figure 2C,D). In addition, the upregulation of circORC2 inhibited the apoptosis of OS and OS DDP‐resistant cells, whereas its downregulation had the opposite effects (Figure 2E).

### 3.3 CircORC2 upregulated TRIM2 expression by targeting miR‐485‐3p

**FIGURE 3:**
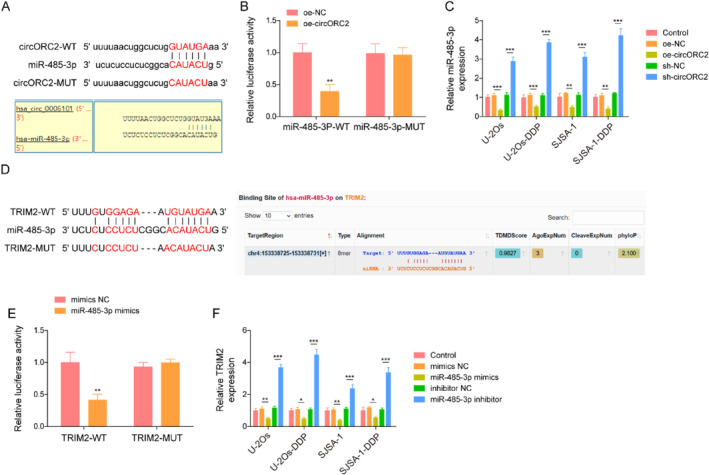
CircORC2 upregulated TRIM2 expression by targeting miR‐485‐3p. (A). The Circinteractome database was used to predict the binding relationship between circORC2 and miR‐485‐3p. (B). The luciferase activity was evaluated via the dual‐luciferase assay. (C). Quantitative reverse transcriptase‐polymerase chain reaction (qRT‐PCR) was employed to detect the miR‐485‐3p level after transfecting the U‐2Os and SJSA‐1 cells with sh‐circORC2 and oe‐circORC2. (D). The starBase database was used to predict the binding sites between miR‐485‐3p and TRIM2. (E). The luciferase activity was evaluated using the dual‐luciferase assay. (F). TRIM2 levels detected by qRT‐PCR after transfecting the U‐2Os and SJSA‐1 cells with miR‐485‐3p mimics and miR‐485‐3p inhibitor. Data are presented as mean ± SD of three replicate experiments (*n* = 3), **p < 0.01 and ***p < 0. 001.

The Circinteractome database was applied to predict the downstream targets of circORC2; targeted binding sites were detected between circORC2 and miR‐485‐3p (Figure 3A). The luciferase activity of miR‐485‐3p‐WT was inhibited by co‐transfection with oe‐circORC2; however, no change in luciferase activity was observed in miR‐485‐3p‐MUT (Figure 3B). The overexpression of circORC2 decreased the miR‐485‐3p level, but the opposite effect was seen with the knockdown of circORC2. The effect was more evident in the OS DDP‐resistant cells (Figure 3C). We predicted the targeting relationship between miR‐485‐3p and TRIM2 from the starBase database and confirmed it using the luciferase activity assay (Figure 3D,E). Similarly, TRIM2 was downregulated in the miR‐485‐3p group and upregulated in the miR‐485‐3p inhibitor group (Figure 3F). These findings indicated that circORC2 upregulated TRIM2 by targeting miR‐485‐3p.

### 3.4 CircORC2 downregulated miR‐485‐3p to promote OS cell proliferation and DPP resistance

**FIGURE 4:**
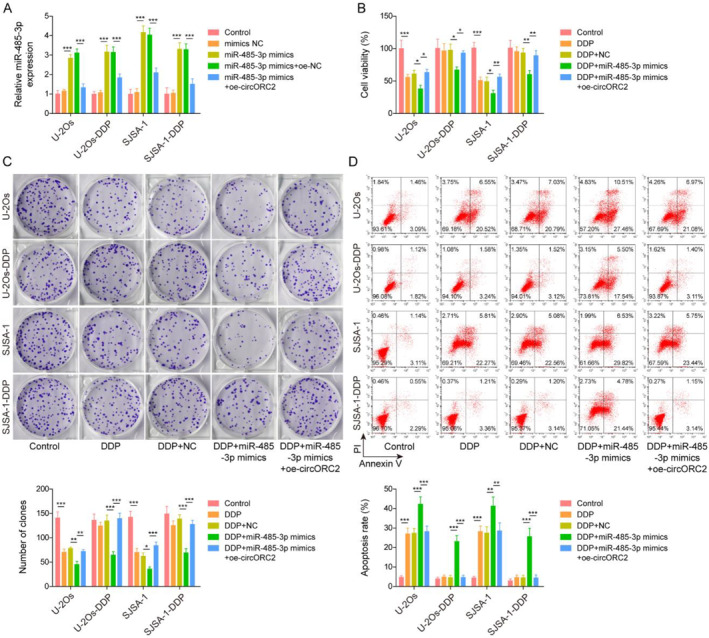
CircORC2 downregulated miR‐485‐3p to promote OS cell proliferation and DPP resistance. U‐2Os and SJSA‐1 cells were transfected with miR‐485‐3p mimics and oe‐circORC2. (A). The miR‐485‐3p level was assessed via Quantitative reverse transcriptase‐polymerase chain reaction. U‐2Os and SJSA‐1 cells were transfected with miR‐485‐3p mimics and oe‐circORC2 and then exposed to DDP. (B). The MTS assay was used to assess the cell viability. (C). Colony formation assay was performed to evaluate cell proliferation. (D). Cell apoptosis was detected by flow cytometry. Data are presented as mean ± SD of three replicate experiments (*n* = 3), *p < 0.05, **p < 0.01 and ***p < 0. 001.

U‐2Os, SJSA‐1 U‐2Os‐DDP, and SJSA‐1‐DDP cells were transfected with oe‐circORC2 and/or miR‐485‐5p mimics. The qRT‐PCR analysis indicated that miR‐485‐3p was highly expressed in OS and OS DDP‐resistance cells after transfection with miR‐485‐5p mimics; these actions were reversed following circORC2 overexpression (Figure 4A). Furthermore, the overexpression of miR‐485‐3p aggravated the inhibitory effect of DDP on cell viability and proliferation but promoted apoptosis; circORC2 overexpression reversed the effects of the miR‐485‐3p mimics (Figure 4B–D).

### 3.5 CircORC2 promoted cell proliferation and DPP resistance by regulating the miR‐485‐3p/TRIM2 axis

**FIGURE 5:**
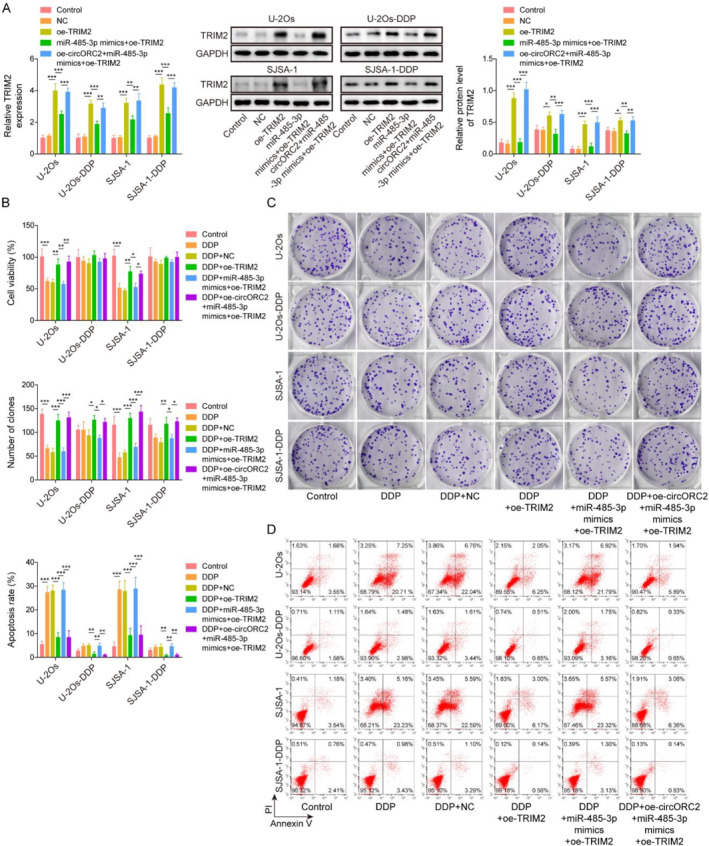
CircORC2 promoted OS cell proliferation and DPP resistance by regulating the miR‐485‐3p/TRIM2 axis. OS cells were transfected with oe‐TRIM2, oe‐TRIM2 with miR‐485‐3p mimics, and/or oe‐circORC2 and then administrated with DDP. (A). Quantitative reverse transcriptase‐polymerase chain reaction was used to detect the TRIM2 level. (B). MTS assay was performed to assess cell viability. (C). Colony formation assay was used to evaluate cell proliferation. (D). Cell apoptosis was detected by flow cytometry. Data are presented as mean ± SD of three replicate experiments (*n* = 3), *p < 0.05, **p < 0.01, and ***p < 0. 001.

The OS DDP‐sensitive and resistant cells were transfected with oe‐TRIM2, oe‐TRIM2 with miR‐485‐3p mimics, and/or oe‐circORC2 and then administrated with DDP. As shown in Figure 5A, the level of TRIM2 was increased in the oe‐TRIM2 group, whereas a decrease in level was observed after transfection with the miR‐485‐3p mimics; the effects of miR‐485‐3p overexpression were reversed following circORC2 upregulation. TRIM2 overexpression inhibited the DDP‐induced reduction in cell viability and proliferation in the U‐2Os, SJSA‐1, U‐2Os‐DDP, and SJSA‐1‐DDP cells, whereas miR‐485‐3p overexpression reversed these effects; however, the upregulation of circORC2 overturned the effects of the miR‐485‐3p mimics (Figure 5B,C). Flow cytometry analysis showed the opposite trend, wherein TRIM2 overexpression inhibited DDP‐induced apoptosis in the OS and OS DDP cells, and the miR‐485‐3p mimics reversed this result; subsequently, circORC2 upregulation reversed the effects of the miR‐485‐3p mimics (Figure 5D). These findings indicated that circORC2 promoted cell proliferation and inhibited the sensitivity of DDP by regulating the miR‐485‐3p/TRIM2 axis.

## 4 DISCUSSION

In recent years, DDP has achieved certain in results OS treatment. However, the overall survival rate of patients with this disease has not improved significantly. The main reason may be the high metastasis and the decrease in the sensitivity of the tumor cells to DDP.^27^ The findings of the present study indicate the role of circORC2 in promoting cell proliferation and inhibiting the sensitivity of OS cells to DDP, thus providing a novel direction for OS treatment.

CircRNA has been used as a biomarker or target in clinical treatments and for disease diagnosis.^28^ Multiple evidence suggests that circRNAs play a key role in developing several diseases by regulating key steps, such as gene transcription, translation, and splicing.^29^ Yang et al. reported that circ_001422 was upregulated in OS tissue and promoted its malignant progression.^30^ Another study reported that inhibition of circRNA_10380 attenuated the proliferation and metastatic ability of OS cells.^31^ In the study by Li et al., circORC2 targeted miR‐19a and enhanced the expression of PTEN to promote the growth of OS cells.^11^ Similarly, the findings of the current study indicated that circORC2 was highly expressed in OS cells and promoted its proliferation, thereby playing a positive role in OS progression. Resistance to chemotherapy is one of the challenges encountered in OS treatment. The circRNA network is thought to play a crucial role in tumor development, including chemotherapy resistance.^32^ Wang et al. illustrated that circPVT1 was overexpressed in OS tissues and cells and promoted chemoresistance via the miR‐24‐3p/KLF8 axis.^33^ Interestingly, the results of the present study indicated that circORC2 inhibited the sensitivity of DDP. Thus, circORC2 appears to play a role in promoting the proliferation and inducing the DDP resistance of OS cells.

There is growing evidence that circRNA regulates gene expression at the transcriptional or post‐transcriptional level through contact with miRNAs or other molecules.^7^ In the present study, circORC2 regulated TRIM2 expression by targeting miR‐485‐3p, an anti‐tumor gene present in various malignant tumors. miR‐485‐3p inhibits malignant proliferation of colorectal cancer cells by targeting TPX2.^26^ Furthermore, miR‐485‐3p was inhibited by circRNA HIPK3 to promote the proliferation and metastasis of renal cancer cells.^34^ Consistently, we found low expression levels of miR‐485‐3p in the OS cells; however, miR‐485‐3p overexpression inhibited OS cell proliferation, indicating that it may play a negative role in OS progression. Furthermore, miR‐485‐3p is involved in regulating tumor drug resistance. Zhao et al. indicated that exosome‐mediated circ_0000338 enhanced 5‐fluorouracil resistance in colorectal cancer by regulating miR‐485‐3p.^16^ LncRNA MALAT1 enhanced paclitaxel resistance by targeting miR‐485‐3p in breast cancer.^35^ In the current study, miR‐485‐3p overexpression enhanced the sensitivity of OS cells to DDP. This is the first study to show the involvement of miR‐485‐3p in regulating DDP resistance in OS.

Additionally, circORC2 promoted cell proliferation and inhibited the sensitivity of OS cells to DDP by regulating the miR‐485‐3p/TRIM2 axis. This study elucidated the targeting relationships between circORC2, miR‐485‐3p, and TRIM2 and the specific mechanisms by which they regulate DDP resistance in OS. TRIM2 is an 81 kDa multidomain protein, also known as CMT2R or RNF86, located at 4q31.3.^36^ It is an important gene involved in the pathogenic process of cancer via different mechanisms and in different microenvironments.^37^ Qing et al. reported that TRIM2 regulates the occurrence and metastasis of OS through the phosphoinositide 3 kinase/protein kinase B signal pathway.^22^ TRIM2 is also associated with chemotherapy resistance; the primary mechanism may be to regulate the process of epithelial‐mesenchymal transformation and obtain the phenotype of cancer stem cells.^38^ Our findings indicated that TRIM2 was upregulated in OS cells. Furthermore, TRIM2 overexpression reversed the inhibitory effects of DDP on cell viability and proliferation and its pro‐apoptotic effects on OS cells. This study elucidated the specific molecular mechanism by which TRIM2 is involved in the regulation of DDP resistance in OS.

In summary, this study showed that circORC2 was upregulated in OS cells and targeted miR‐485‐3p, which in turn targeted TRIM2. circORC2 promoted cell proliferation and inhibited the sensitivity of OS cells to DDP via the miR‐485‐3p/TRIM2 axis. Thus, circORC2 may be an effective target to disrupt the resistance of tumor cells to DDP.

## AUTHOR CONTRIBUTIONS

**Tianhua Chen**: Conceptualization, methodology, writing–original draft preparation, investigation, validation, visualization. **Zuyang Zhang**: Methodology. **Chao Tian**: Data curation. **Yuchao Feng**: Investigation. **Xiaojie He**: Data curation. **Liangdong Jiang**: Conceptualization, writing–original draft preparation, supervision, writing–reviewing and editing.

## CONFLICT OF INTEREST STATEMENT

There are no conflicts of interest to declare.

## ETHICS STATEMENT

This study did not involve animal or clinical trials, so the Ethics Statement is not applicable in this study.

## Supporting information

Figure S1

## Data Availability

The raw data supporting the conclusions of this manuscript will be made available by the authors, without undue reservation, to any qualified researcher.
