# Supplementary material for: CircORC2 promoted proliferation and inhibited the sensitivity of osteosarcoma cell lines to cisplatin by regulating the miR‐485‐3p/TRIM2 axis
Source: J Cell Commun Signal. 2024 Apr 25;18(2):e12029. doi: 10.1002/ccs3.12029 (PMC11208123; doi:10.1002/ccs3.12029)
Supplement: Supplementary file 1 — Figure S1 [file CCS3-18-e12029-s001.doc]

Supplementary materials


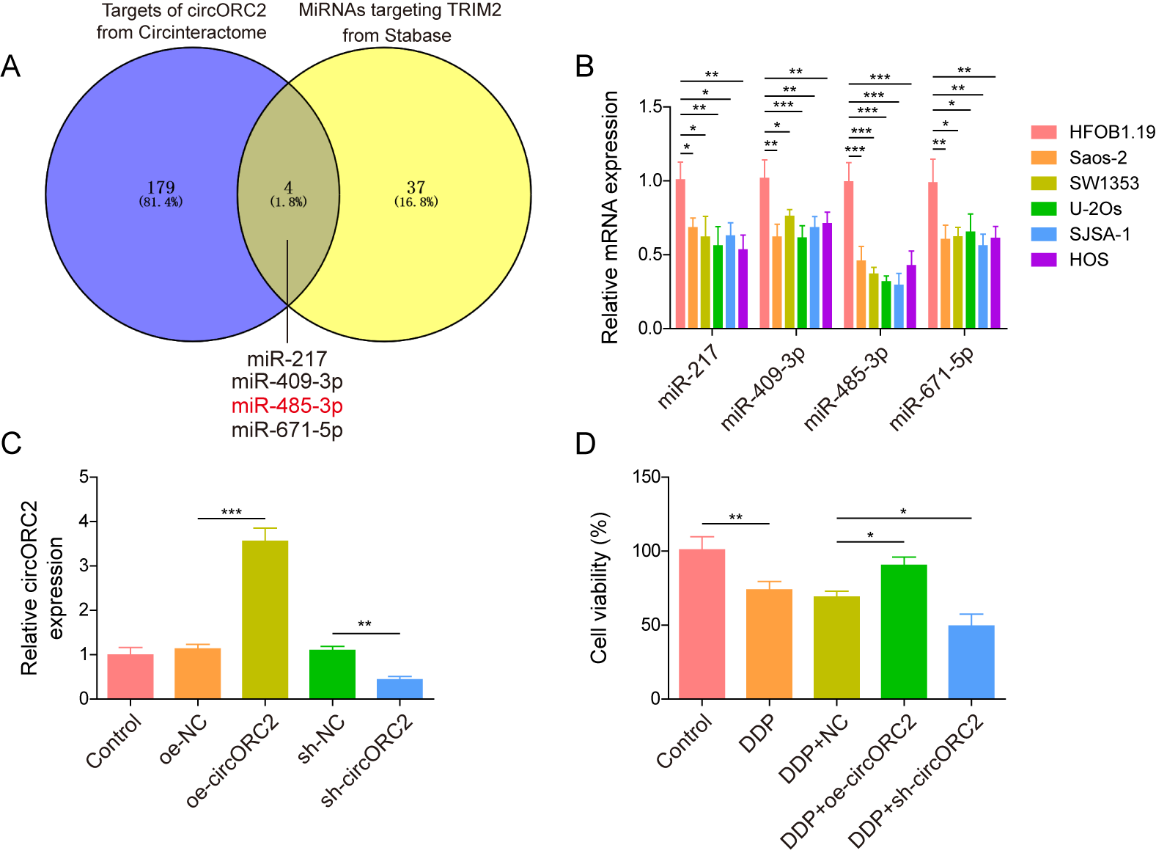


**Figure S1.** **The selection of miR-485-3p and the effect of DDP on circORC2 overexpression and knockdown OS cell viability.** A. Venn diagram of the target miRNAs of circORC2 from the CircInteractome database and target TRIM2 from the starBase database. B. qRT-PCR was performed to detect the miRNA levels in circORC2 level in the OS cell lines and HFOB1.19 cell. C. Overexpression or knockdown of circORC2 in the HFOB1.19 cells, and circORC2 expression was detected by qRT-PCR. D. MTS assay was performed to examine the cell viability.
